# Clinical, Radiologic, and Functional Outcomes following Bone Grafting for Metacarpal Non-Unions: A Systematic Review

**DOI:** 10.3390/jcm13041148

**Published:** 2024-02-18

**Authors:** Omar El Sewify, Jad Abi-Rafeh, Jack Legler, Shayan Karimi, Aslan Baradaran, Johnny I. Efanov

**Affiliations:** 1 Faculty of Medicine, Laval University, Quebec, QC G1V0A6, Canada; 2 Plastic, Reconstructive and Aesthetic Surgery, Department of Surgery, McGill University, Montreal, QC H3G2M1, Canada; 3 Faculty of Medicine and Health Sciences, McGill University, Montreal, QC H3G2M1, Canada; 4 Plastic and Reconstructive Surgery, Department of Surgery, Centre hospitalier de l’Universite de Montreal (CHUM), Montreal, QC H2X3E4, Canada

**Keywords:** metacarpal, non-union, bone graft

## Abstract

**Objectives**: Metacarpal non-unions are complex hand defects that can lead to severe hand impairment. Treatment may require the use of artificial or autologous bone grafts. This systematic review aims to describe the outcomes of bone grafting following metacarpal non-union in an attempt to establish an optimal therapeutic protocol for this complication. **Methods**: A systematic review was conducted in adherence with PRISMA guidelines. Data collection and analysis were performed in duplicate and confirmed by a third investigator. Our primary outcomes focused on radiological time to bone fusion and rates of non-union. Additionally, functional outcomes and complications were analyzed as means of central tendency. **Results**: Eighteen studies were included in the systematic review, accounting for a total of 47 patients. The average follow-up time was 12.4 months. Fourteen studies analyzed radiological outcomes, with atrophic non-union representing the most common type. The time to bone fusion, assessed radiologically, following bone graft was an average of 6.9 months (n = 14), with a 100% rate of union in 42 patients. Regarding patient-reported pain improvement, 76% of patients experienced pain relief. Moreover, all patients reported a complete subjective return to baseline hand function. Adverse events, limited to hematoma and seroma, were seen in three patients, representing a complication rate of 11.8% in the examined population. **Conclusions**: Metacarpal non-union can be treated successfully via vascularized and non-vascularized bone grafting. Based on the available evidence, bone grafts demonstrate favorable union rates, post-operative pain reduction, hand function recovery, earlier bone fusion times, and minimal complications in the context of metacarpal non-union management.

## 1. Introduction 

Non-union is defined by a bone being unable to heal for a minimum of six months following injury and no signs of improvement for three successive months [1,2,3]. The estimated prevalence of all non-unions ranges between 1.9 and 10%, and in the United States alone, approximately 100,000 fractures develop into non-unions annually [4,5]. In addition to patient-dependent risk factors, the discrepancy in prevalence may be attributed to the anatomical region of the fracture [6,7,8,9,10,11]. The presence of open fractures and soft-tissue injuries has also been reported to increase the occurrence of non-unions by up to 16% [12,13].

Metacarpal non-union is a unique hand injury that requires immediate intervention to prevent severe functional impairment [14,15]. As a non-union remains untreated, pain, stiffness, reduced range of motion, and impaired function progress in tandem [16,17]. The exacerbation of these symptoms and their impact on patients’ functioning and activities of daily living (ADLs) can place significant weight on their mental health and hinder quality of life [18].

The initial treatments for non-unions remain conservative, with longer term cast immobilization and various bone stimulators such as low-intensity ultrasound representing viable options [5]. To this end, the novel conservative treatments, such as pulse electromagnetic field therapy, have significantly improved bone consolidation in the context of acute distal radius fractures [19]. However, surgical treatment may be necessary when conservative measures fail to provide symptomatic relief or progressive functional healing. In the absence of widely embraced guidelines delineating the failure of conservative management, various studies have identified the requirement for surgical intervention in delayed union and non-union based on the absence of bone consolidation after three and six months, respectively [1,2].

A recent review on the definition of metacarpal non-unions concluded that the characterization of metacarpal non-union is highly variable and lacks a standardized definition [20]. Traditionally, surgical approaches have involved the use of percutaneous or internal fixation, bone grafts, or a combination of both in an effort to reach mechanical stability and facilitate bone healing [5,21]. As of late, emerging evidence within the orthopedic literature has demonstrated bone grafting to be an effective treatment option for long bone and carpal bone non-unions [3,14,22]. Within the realm of plastic surgery, non-vascularized bone grafts have been extensively used for the treatment of scaphoid non-union [3]. Given the lack of reported metacarpal non-unions, their incidence within a broader population remains unknown. Hence, evidence-based insight into optimal management remains essential [20]. Nevertheless, the plethora of grafting techniques has made it difficult for the surgical community to reach a consensus gold standard for the treatment of non-union hand fractures [22]. Gaining an in-depth understanding of the most effective treatment approaches is crucial in paving the way toward a widely adopted treatment protocol for metacarpal non-unions amongst this patient population. 

In comparison with the numerous studies on the outcomes of bone grafting for the management of scaphoid non-unions, very little has been reported on the use of bone grafting in the management of metacarpal non-unions. As such, the following systematic review aims to describe radiological and functional outcomes following bone grafting in metacarpal non-unions and provide recommendations on an optimal approach. 

## 2. Methods

### 2.1. Search Strategy

A systematic review was conducted in accordance with the Preferred Reporting Items for Systematic Reviews and Meta-Analyses (PRISMA) guidelines [23]. A protocol for this study was registered on Open Science Framework (https://osf.io/egx3a, accessed on 1 March 2023)*,* and a search was subsequently performed from the study’s inception through June 2023 using Medline (via Ovid), Embase (via Ovid), and Cochrane Library. Search strategies are provided in Supplementary Tables S1.1–S1.3.

### 2.2. Study Selection and Eligibility

English prospective, retrospective, randomized controlled trials, case series, case–control studies, and case reports were eligible for inclusion. Systematic reviews, meta-analyses, letters to the editor, viewpoints, commentaries, cadaveric studies and abstracts that could not be traced back to the full text were excluded. Studies reporting on either adult or pediatric patients with or without a comparator group were included. References were uploaded to Rayyan (a web and mobile app for systematic reviews). Title, abstract screening, full text review, and data extraction were performed in duplicate by reviewers (SK and JL). Any disagreements were resolved through discussion with a third reviewer (OE). Microsoft Excel (Microsoft Corp., Redmond, WA, USA) was used for data extraction and analysis. Supplementary Table S2 provides the reasons for exclusion for the studies excluded at the full text review stage. The data points we extracted are provided in Supplementary Table S3. Extracted data are provided in summary tables, and where applicable, descriptive statistics are presented (i.e., complication rates and overall means).

## 3. Results

### 3.1. Study Characteristics

**Figure 1:**
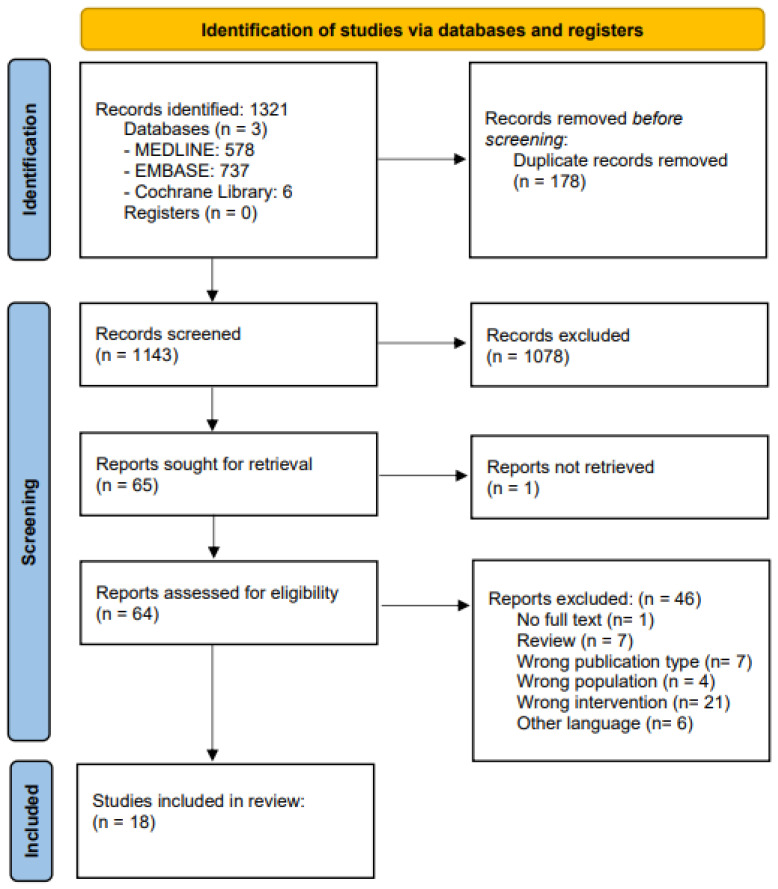
PRISMA flow chart for identification of studies via databases and registers.

The initial search identified 1321 articles. Following de-duplication and the removal of 178 studies, 1143 articles were screened based on their titles and abstracts. A total of 64 articles were screened through a full text review, and 18 were identified to satisfy the inclusion criteria (Figure 1).

The studies included three prospective cohorts, two retrospective cohorts, two case series, and eleven case reports, reporting on a total of 47 patients (Table S4). 

### 3.2. Patient Characteristics

Among the pooled patient cohort examined, 83% were males (n = 39), and 17% were females (n = 8). The mean age was 30.2 years (range: 2–60), and 41.7% of patients had their dominant hand affected (n = 7). Sixteen studies reported on an initial metacarpal fracture, with the third metacarpal being the most affected (n = 9), followed by both the fourth (n = 5) and fifth metacarpal (n = 5). Five studies reported on the nature of the injury. Half (n = 5) suffered an open fracture, whereas the remaining half suffered a closed fracture (n = 5). Four studies with a total of 11 patients described the fracture location. Eight fractures were sustained at the metacarpal shaft, two fractures occurred at the base, and one was located at the neck. Overall, seven patients across six studies were treated initially via open reduction and internal fixation (ORIF) utilizing plate fixation and screws. One patient was treated via ORIF in conjunction with an autologous distal radius bone graft. Four articles used ORIF in isolation, and one article described a reconstruction with stump coverage using pedicled groin flaps. Moreover, one patient was subject to debridement prior to non-union. Four articles (n = 8) used K-wires as an initial intervention for the metacarpal fracture, whereas three articles (n = 3) opted for plates and screws. Two articles (n = 4) used plates in isolation, and one article (n = 1) used headless screws as a primary intervention. Finally, two patients across two different articles were initially treated with splints, while another article (n = 1) used a Steinmann pin. The average number of operations prior to grafting was 1.3 (n = 16) (Tables S5.1 and S5.2).

### 3.3. Non-Union Features and Grafting Techniques

Seven articles reported an atrophic non-union, which represented the most common type of non-union. Only 1 article reported a hypertrophic non-union, and 14 articles did not describe or specify the type of non-union. In terms of the etiology of the non-union, 8.9% were attributed to infection. The mean time from injury to grafting and mean time from the last intervention were 9.7 months (n = 15) and 4.5 months (n = 10), respectively. 

Various bone grafting techniques were described and implemented either in isolation or in conjunction with ORIF. Sixteen articles described the type of bone graft used, and all reported using a variation of autogenous bone graft, except for one patient who underwent an allograft cancellous bone graft. Five articles (31%) implemented vascularized bone grafts, whereas eleven articles (69%) used non-vascularized bone grafts. Amongst the latter, four (36%) described cancellous bone grafts, three (27%) described cortical bone grafts, and the remaining four (36%) did not provide details on the specific type of bone utilized. Two articles omitted details regarding the grafting technique employed. Twelve articles used a combination of grafting and ORIF. The donor sites used include the radius (n = 2), iliac crest (n = 8), and femur (medial condylar [n = 4] and supracondylar area [n = 2]) (Tables S5.1 and S5.2). 

### 3.4. Post-Operative Outcomes

The average time to bone fusion following bone graft was 6.9 months, and a 100% rate of union was achieved in 42 patients (n = 14). The average mean post-operative immobilization time was 1.7 weeks (n = 8), and the post-operative immobilization time ranged from 0 to 3 months. The overall mean time to follow-up was 12.4 months (n = 16, mean range: 1.5–60 months). Fourteen articles reported on radiologic outcomes; twelve articles reported on functional outcomes, such as range of motion (n = 11), grip strength (n = 5), and pinch strength (n = 3). Four articles described patient-reported outcomes, including visual analogue scale scores (n = 1), patient sensation (n = 4), and patient satisfaction (n = 3). In fact, 65% of patients stated that they were satisfied with the results of the procedure. Among the eight articles reporting on pain improvement, 76% of patients experienced pain relief, and seven articles reported 100% improved functionality following bone grafting. Adverse events were only observed in three patients, representing 11.8% of the reported population (n = 26). Among the studies reporting on a return to ADL (n = 10), fifteen patients reported subjectively returning to their baseline function, with the average time to return being 4.5 months. 

Non-vascularized bone grafts (NVBGs) emerged as the predominantly assessed treatment for metacarpal non-union, with outcomes being detailed for 32 patients. The mean time to bone fusion in NVBG patients was 6.3 months, while vascularized bone graft (VBG) patients demonstrated a longer mean time of 8.6 months. As such, based on the data gathered, both modalities yielded successful union and favorable outcomes. Notably, non-vascularized bone grafts demonstrated a shorter time to bone fusion compared to their vascularized counterparts. Our data further indicate that the incorporation of any bone graft technique in tandem with surgical fixation is more effective than relying solely on internal fixation (Table 1 and Table 2). For a comprehensive overview of all pooled data, please refer to Table 3 and Table 4.

## 4. Discussion

This systematic review aimed to evaluate the post-operative outcomes of bone grafts in the treatment of metacarpal non-unions with the goal of providing a recommendation on an optimal treatment approach. Eighteen papers reporting on the treatment of 47 patients were included and analyzed herein. The overall mean follow-up time was 12.4 months. The average time for fusion following bone graft was 7.4 months, with a 100% rate of union being observed in 42 patients. Post-operative pain was not reported by any patient during follow-up, and all patients returned to their prior activities of daily living. Only three complications were noted: one donor-site hematoma, one recipient-site hematoma, and one recipient-site prolonged seroma. In summary, these findings demonstrate that the utilization of bone grafts is a safe and practical surgical technique for treating metacarpal non-unions.

Management strategies for metacarpal fractures frequently hinge on the surgeon’s preference and expertise, introducing wide variability in treatment approaches [24,25,26,27]. Other factors, such as comorbidities, age, fracture location, and nature of injury, play an important role in determining the most effective course of treatment [28,29]. 

The timing of an intervention post-hand fracture is imperative, as delays can have a significant impact on both patient functionality and productivity [30,31]. Up to 1.5% of metacarpal fractures result in non-union [20,32]. In fact, metacarpal non-union is more prevalent following open injuries associated with periosteal stripping and soft tissue trauma [33]. Injuries of this nature can result in segmental bone loss or neurovascular compromise, which hinders the natural bone healing process [34,35]. Infection secondary to the initial trauma or hardware introduction may also cause poor bone consolidation, ultimately leading to metacarpal non-union [35]. Other risk factors include bone fragment overdistraction following external fixation or vascular compromise following periosteal stripping required during ORIF [33,36].

Hayes et al. reported on the definition of metacarpal non-union across different investigations in the literature [20]. They concluded that the characterization of metacarpal non-union is highly variable and lacks a standardized definition. As such, the absence of a standardized definition makes it challenging to develop clear clinical guidelines for the management of metacarpal non-union, potentially affecting the quality and consistency of patient care.

While guidelines advise using internal over external fixation in the treatment of metacarpal non-union, both approaches can be employed synergistically alongside bone grafts in accordance with a surgeon’s preference [37,38]. Although internal fixation may result in extensor lag or stiffness, it allows for adequate alignment and bone stability [38]. On the other hand, fixation techniques like external fixation or K-wires require hardware removal and carry a risk of infection at the fixation sites [38]. In Aguilera et al.’s study, 50% of patients (n = 2) underwent non-vascularized bone grafting alongside ORIF, while the remaining half underwent internal fixation alone (n = 2) [39]. The latter group achieved bone fusion at 10 weeks. In contrast, the combined treatment group had one patient achieve fusion at 45 days and another at 5 weeks. To this end, the current recommendations for metacarpal non-union suggest the use of bone grafts to repair the bony deficit in conjunction with open reduction and internal fixation [15,22,40]. However, the supporting evidence for these guidelines in the clinical setting is limited [39]. 

The efficacy of bone grafts in treating non-unions in various anatomical locations has been corroborated by other studies in the literature [41,42]. This provides valuable insights for developing an optimal treatment guideline for metacarpal non-unions. For example, in the management of humeral non-union, Choudry et al. demonstrated that bone grafting led to union in 95% of cases [41]. Similarly, Ferguson et al. reported union rates of 80% and 84% for scaphoid non-unions treated with non-vascularized and vascularized bone grafts, respectively [42]. Hirche et al. further demonstrated that patients who underwent NVBG in the treatment of scaphoid non-union achieved a higher union rate (82.2%) than those subjected to VBG (75%) [43]. Compared to VBGs, NVBGs are considered to be technically less challenging, have shorter surgery times and decreased donor site morbidity, and allow for better bone graft adaption to the anatomy of the recipient site [44,45]. Despite these promising results, it is important to note that both humoral and scaphoid fractures have distinct mechanisms of injuries compared to metacarpal fractures in addition to contrasting baseline anatomical differences that impact the utility of VBGs and NVBGs. The most common bone grafting donor sites for vascularized and non-vascularized bone grafts are the medial femoral condyle and iliac crest, respectively [33,46,47]. This was further supported by the data collected for this review, as we found that the iliac crest emerged as the predominant donor site for NVBGs. On the other hand, both medial and supracondylar femoral areas were most frequently utilized as donor sites for VBGs.

In our review, a total of 19 patients across five different studies received VBGs. While successful union was achieved in these cases, it is important to note that similar outcomes have been observed in patients who received NVBGs. While both techniques are viable options in the management of metacarpal non-union, based on the current literature, along with data gathered in this review, NVBGs are associated with reduced time to bone fusion [45,48,49]. However, additional research is warranted to differentiate the effectiveness of these bone graft techniques in different clinical settings. 

Various types of bone grafts may also be applied depending on the size and extent of the wound. Defects characterized by inadequate vascular supply to the adjacent soft tissue, infected fractures, or secondary cases after unsuccessful reconstruction with non-vascularized grafts also require vascularized bone grafting [50,51,52]. Traditional non-vascularized bone grafts are not recommended in cases of poorly vascularized bone beds [38]. The current literature describes a bone gap greater than 6 cm to be an indicator for the use of a vascularized bone graft [50,51,52]. Allsopp et al. further investigated the evidence surrounding the 6 cm rule and concluded that there was no clear evidence supporting the need for vascularized bone grafts for longer defects [45]. In fact, most of the articles examined by the authors presented inconclusive evidence regarding the correlation between defect length and vascularization requirements. These articles also demonstrated that NVBGs are not recommended for bone defects greater than 5–7 cm [45,53,54,55]. This was corroborated by Pogrel et al. in their comparative study of mandibular defects [56]. The authors of this particular study demonstrated that an increased failure rate of NVBGs corresponded with an increased defect length. The absence of compelling evidence investigating the association between graft length and the optimal bone graft technique underscores a current clinical gap, highlighting the need for future research to address this significant area. 

The intricate realm of non-union hand fractures necessitates an optimal post-operative care regimen—a pivotal phase of the rehabilitation process. An appropriate post-operative treatment plan is crucial, not only for preventing complications but also, more importantly, for reinstating anatomical integrity and hand function. Current evidence advocates for hand therapy and the post-operative immobilization of fractured carpal, metacarpal, or phalanx bones through the application of a cast or splint [5,43,47,57]. Although immobilization helps facilitate proper healing and prevent additional stress applied onto the graft, prolonged immobilization can impact a patient’s range of motion and lead to complications, including tendon adhesion and joint stiffness [33,39]. Hirche et al. required their patients to remain immobilized in their cast for a minimum of 6 weeks until union was achieved [43]. Similarly, Kim et al. had patients immobilized in a cast for 6 to 8 weeks followed by the provision of a splint to facilitate low-risk hand exercises once the cast was removed [47]. Tsumura et al. proposed an immobilization period of seven weeks or less for scaphoid non-union after VBG considering no complications or graft failure [47]. In fact, the authors found that immobilization for greater than seven weeks resulted in a decreased range of motion compared to immobilization for less than seven weeks. The present review provides additional support for post-operative immobilization and physical therapy with an average immobilization duration of 1.7 weeks (range 0–3 months), and no decreases in range of motion among patients were observed. 

Bone growth stimulation (BGS) use has been proven to promote bone healing as an adjuvant to bone grafting [5,57,58,59,60]. In a recent review, it was reported that low-intensity pulse ultrasound (LLPU) achieved an overall union rate of 82% across different anatomical locations [61]. Furthermore, within a subgroup analysis focusing on scaphoid non-unions, a union rate of 78% was achieved. Similarly, Haller et al. investigated the effectiveness of LLPU as a first-line treatment for long bone fractures [62]. They reported a union rate of 94%, with only one patient requiring further surgical intervention. LLPU treatments are carried out through a portable device that is programmed to deliver daily 20 min ultrasound sessions, meaning that treatment can be self-administered by the patient in the comfort of their own home. LLPU treatment regimens can last up to 6 months depending on the nature and severity of the fracture [63]. The aforementioned studies all employed LLPU as a non-operative treatment modality in isolation. However, LLPU use favors the shortening of healing times. Thus, considering its favorable outcomes in bone healing, it may be effective as an adjuvant to bone grafting [57,59,60]. 

The primary goal in treating metacarpal non-union is to return patients to their baseline level of ADL. However, various factors, such as surgical complications and post-operative pain, may hinder the achievement of this objective. Joint stiffness, infection, and persistent non-union or malunion are common adverse events that patients may experience following bone grafting procedures [14]. Although few patients included in our review encountered these setbacks, approximately 7.7% faced other complications after surgery. Nevertheless, it was not reported whether these individuals face a prolonged time to bone fusion compared to the mean of 7.4 months. Furthermore, despite these adverse events, the cohort did not report any post-operative pain at follow-up.

Our systematic review was not without limitations. Firstly, there was a lack of consensus regarding definitions of non-union versus delayed union. In this systematic review, only non-unions of more than 6 months were selected. Moreover, the included studies had a predominantly homogenous methodology, with most articles being case reports. Consequently, the true impact of bone grafting in the treatment of metacarpal non-union may be overestimated due to the nature of case studies and the absence of comparative groups. Additionally, the small sample size may have led to an underestimation of complications. Secondly, an inherit language bias was introduced, as only articles written in English were considered. Thirdly, different permutations of initial injuries, preliminary intervention strategies, time from injury to graft, and bone graft types were applied in the included studies. Thus, the achievement of metacarpal union may be influenced by these external factors. Finally, the lack of evidence and the variability of the outcomes reported in each study made it challenging to directly compare NVBGs and VBGs. In turn, it was difficult to establish a conclusive and universally applicable treatment protocol. Nonetheless, our findings demonstrate that the utilization of bone grafts is a safe and practical surgical technique for treating metacarpal non-unions.

## 5. Conclusions

Herein, we reported that metacarpal non-union can be treated successfully via either NVBGs or VBGs. Yet, the radiological and clinical efficacy of these techniques remains limited. Existing evidence suggests that the use of both modalities in conjunction with ORIF, LLPU, and an appropriate period of immobilization (<7 weeks) confer superior outcomes rather than using either of them in isolation. Both grafting techniques provide favorable outcomes in terms of union rates, post-operative pain reduction, and hand function recovery, leading to minimal complications. However, NVBGs shorten the time to bone fusion. Therefore, the use of bone grafts can be considered a modality of choice for defects pertaining to the metacarpal. Nevertheless, additional, larger randomized controlled trials are required to thoroughly investigate post-operative outcomes and complications. We recommend that future research on metacarpal non-unions employ standardized outcome measures in order to account for homogeneity amongst trials, facilitating improved data aggregation.

## Figures and Tables

**Table 1:** Postoperative outcomes.

Study	Management	Time to Follow up (Mean)	Time to Bone Fusion (Mean)	Rates of Non-Union (%)	Radiological Outcomes	VAS Score	Pain	ROM	Grip Strength
Aguilera et al. (2022)	M1: Immobilized for 2 w M2: Immobilized with wrist plaster for 7 d, and finger buddy taping for 3 w M3: NR F1: NR	M1: 5 w M2: 45 d M3: 10 w F1: 2 m (1.8 m)	M1: 5 w M2: 45 d M3: 10 w F1: NR (1.8 m)	0/3 (0%)	M1: Bone healing at 5 w postoperatively M2: Fracture healing present at 45 d of evolution M3: Complete bone healing after 10 w F1: NR	NR	M1: NR M2: NR M3: NR F1: Completely painless	M1: Same as contralateral side M2: Full ROM at 45 d of evolution M3: Comparable to contralateral side. F1: Full ROM without any fracture mobility	M1: NR M2: NR M3: NR F1: 14 kg vs. 16 kg in the uninjured side
Akmaz et al. (2004)	Wound irrigation and debridement	M1: 12 m M2: 13 m M3: 15 m M4: 20 m M5: 8 m M6: 30 m M7: 9 m M8: 11 m (14.8 m)	M1: 16 w M2: 10 w M3: 12 w M4: 12 w M5: 8 w M6: 16 w M7: 10 w M8: 12 w (12 m)	0/8 (0%)	Radiological union established in all cases	NR	All fingers were painless	Pre: Post (MP joint) M1: 2nd metacarpal 40°:75° 3rd metacarpal: 30°: 60° M2: 55°:75° M3: 40°:85° M4: 60°:60° M5: 50°:80° M6: 50°:80° M7: 60°:80° M8: 55°:85°	Pre (injured side/uninjured side): Post (kg) M1: 16/35:20 M2: 20/35:26 M3: 15/42:19 M4: 22/35:27 M5: 20/38:25 M6: 20/35:24 M7: 20/38:23 M8: 20/40:26
Anderson et al. (2022)	Maximal hand therapy	Short-term: 2 y post-injury Long-term: 5 y post-injury	NR	NR	NR	NR	2y post-injury: Discomfort at the dorsal metacarpal plate 5y post-injury: Overall doing well	MPJ: - 10° to 80° PIPJ: +15° to 100° DIPJ: 0° to 65° TAM: 270°	41 lbs vs. 110 lbs in uninjured hand
Christen et al. (2022)	100 mg Aspirin DIE × 1 m	NR	P1: 7 m P2: 5 m P3: 7 m P4: 8 m (6.75 m)	0/4 (0%)	NR	NR	NR	P1: Severe 5th ray stiffness P2: Full active ROM, slight 15° PIPJ extension lag P3: Limited 60° PIPJ flexion P4: Limited 25° MPJ flexion, MPJ extension stiffness	NR
Cogsil et al. (2022)	Hand therapy and removable brace started at 1 w, worn ×6–8 w based on radiographic bony union evidence	M: 3 m F: 4 m (3.5 m)	M: 3 m F: 4 m (3.5 m)	0/2 (0%)	M: Solid healing at 3 m postoperatively F: Full consolidation and healing 4 m postoperatively	NR	M: NR F: Pain free	M: Full finger motion F: NR	Pre/Post (lb) M: 125/50 F: NR
Deng et Al. (2020)	NR	24 m	24 m	0/1 (0%)	United graft with no signs of instability	NR	Pain free	70° MPJ flexion, no extension lag	NR
Doi and Sakai (1994)	NR	NR	2m	0/1 (0%)	NR	NR	NR	NR	NR
Ebraheim et al. (1997)	NR	1st FU: 3 m 2nd FU:12 m	3 m	0/1 (0%)	NR	NR	NR	Full digital flexion/extension at 12 m	NR
Erçin et al. (2022)	Postoperative IV fluids, analgesics, antibiotics & anticoagulants, Volar splint applied, and flaps checked every 4 h	6 m	6 m	0/11 (0%)	Union at 6 m for all patients	P1 (M): 3 P2 (M): 3 P3 (M): 4 P4 (F): 3 P5 (M): 4 P6 (M): 3 P7 (M): 4 P8 (F): 7 P9 (M): 3 P10 (M): 5 P11 (M): 4	NR	NR	NR
Ferguson and Bogoch (1999)	Hand splint for 3 m postoperatively and then casted	27 m	27 m	0/1 (0%)	Graft incorporation observed at 27 m	NR	Pain free	0° to 70° active MPJ ROM	NR
Ireland and Taleisnkik (1986)	Short arm cast immobilization for 6 w postoperatively	First FU: 2 m Second FU: 3 m	3 m	0/1 (0%)	Non-union healed 3 m postoperatively	NR	NR	NR	NR
Jupiter et al. (1985)	NR	NR	P1: 12 w P2: 12 w P3: 8 w P4: 12 w P5: 12 w P6: 8 w (2.7 m)	0/6 (0%)	NR	NR	NR	Pre: Post P1: MPJ: 0/90°: 0/90° TAM: 250°:250° P2: MPJ: 0/10°:0/70° TAM: 250°:250° P3: MPJ: 0/90°:0/90° TAM: 190°:200° P4: MPJ: 0/45°:0/65° TAM: 170°:210° P5: 2nd metacarpal MPJ: 0–90°:0–90° TAM: 250°:250° P6: MPJ: 0/50°:0/80° TAM: 195°:245°	NR
Milhoan et al. (2022)	Ulnar gutter splint immobilization for 2 w	1st FU: 2 w 2nd FU: 6 w 3rd Fu: 6 m	NR	NR	NR	NR	Pain resolution at 2 w FU	Recovered full active and passive digital motion at 6 m	NR
Sakai et al. (1988)	NR	7 m	2 m	0/1 (0%)	NR	NR	NR	NR	NR
Tsai et al. (1981)	NR	43 m	NR	NR	NR	NR	NR	2nd toe (index finger): ML: 50/65° PIPJ: 40/45° DIPJ: 15/30° 3rd toe (little finger): ML: 40/65° PIPJ: 30/60° DIPJ: 20/45°	26 lb
Vegas et al. (2012)	NR	9 m	9 m	0/1 (0%)	Unequivocal bone union signs at 9 m	NR	NR	NR	NR
Wei et al. (1999)	NR	57 m	NR	NR	NR	NR	NR	NR	NR
Zargarbashi et al. (2018)	Active motion & physical therapy as soon as possible and K-wires removal at 6 w postoperatively	1.5 m	6 m	0/1 (0%)	Healed 6 m post-treatment	NR	NR	NR	NR

DIPJ: Distal interphalangeal joint; F: Female; FU: follow-up; IV: Intravenous; lb: Pounds; M: Male; m: months; ML: Metatarsophalangeal joints; MPJ: Metacarpophalangeal joint; NR: Not reported; N: Number of participants; P: patient; PIPJ: Proximal interphalangeal joint; TAM: Total active motion; w: weeks; y: years.

**Table 2:** Postoperative Outcomes Continued.

Study	Pinch Strength	Surgical Complications	Return to ADLs	Patient Satisfaction	Sensation
Aguilera et al. (2022)	NR	Zero	M1: NR M2: Returned to everyday unrestricted activities M3: NR F: NR	NR	NR
Akmaz et al. (2004)	NR	Pintract infection in 1 patient, treated successfully with wound dressings and oral antibiotics	All able to use their hands for daily activities postoperatively	NR	Adequate circulation and sensation in all fingers
Anderson et al. (2022)	NR	Zero	Capable of performing ADLs and associated hobbies at 5 y postoperatively	Overall pleased	6/10 at digit tip
Christen et al. (2022)	NR	Zero	NR	P1: NR P2: NR P3: Satisfied P4: NR	NR
Cogsil et al. (2022)	M: Pre/Post (lb): 22/11 F: 10 lb vs. 22 lb contralaterally	Zero	NR	NR	NR
Deng et Al. (2020)	NR	NR	NR	NR	NR
Doi and Sakai (1994)	NR	NR	NR	NR	NR
Ebraheim et al. (1997)	NR	NR	Return to previous occupation at 12 m	NR	NR
Erçin et al. (2022)	NR	Donor site hematoma: 1 Recipient site hematoma: 1 Recipient site prolonged seroma: 1	NR	P1 (M): 3, P2 (M): 4 P3 (M): 3, P4 (F): 3 P5 (M): 4, P6 (M): 4 P7 (M): 3, P8 (F): 4 P9 (M): 3, P10 (M): 4 P11 (M): 4 (1 = very bad, 5 = excellent)	NR
Ferguson and Bogoch (1999)	NR	NR	Carry out work/leisure activities	NR	NR
Ireland and Taleisnkik (1986)	NR	NR	NR	NR	NR
Jupiter et al. (1985)	NR	NR	NR	NR	NR
Milhoan et al. (2022)	NR	Zero	Cleared to resume normal activities at 6 w postoperatively	NR	NR
Sakai et al. (1988)	NR	Zero	NR	NR	NR
Tsai et al. (1981)	Between thumb & 2nd toe/index finger: 9 lb Between thumb & 3rd toe (little finger): 6.5 lb	NR	Returned to former job in grain elevator	NR	Two-point discrimination: 2nd toe/index finger: 15 mm 3rd toe/little finger: 12 mm
Vegas et al. (2012)	NR	NR	NR	NR	NR
Wei et al. (1999)	Pulp-to-pulp pinch on L hand: 2.5kg Tripod pinch power on R hand: 3.6 kg	Zero	Returned to previous activities and job as a guard, uses a gas pistol and drives a motorcycle to work.	NR	Moving two-point discrimination at 57 m FU: Thumbs: 4-mm 3 new fingers: range of 4 to 1 mm
Zargarbashi et al. (2018)	NR	Zero	NR	NR	NR

F: Female; FU: Follow up; kg: Kilograms; L: left; lb: Pounds; M: Male; m: months; NR: Not Reported; N: number of participants; P: patient; R: right; w: weeks; y: years.

**Table 3:** Pooled Analysis.

	Mean Age (years)	% of Patients with Dominant Hand Affected	Mean Prior # of Bone Operations	Mean Time from Injury to Graft (months)	Mean Time from Last Intervention (months)	% Atrophic Nonunions	% Nonunions due to Infection	Mean Time to Follow-Up (months)
**Value**	30.2	41.7	1.3	9.7	4.5	50	8.9	12.4
**N Studies**	18	7	16	15	10	7	16	16

m: months; w: weeks; y: years; #: number.

**Table 4:** Pooled Analysis continued.

	Mean Postoperative Immobilization Time (Weeks)	Mean Time to Union (Months)	% of Graft Failures	% of Pain Improvement	% of Complications	% of Patients Return to ADLs	% Patient Satisfaction Rate	% of Improved Functionality
**Value**	1.7	6.9	0	76	11.8	4.5 m	61.5	100
**N Studies**	8	14	14	8	10	10	3	7

ADLs: Activities of daily living.

## Supplementary Materials

The following supporting information can be downloaded at: https://www.mdpi.com/article/10.3390/jcm13041148/s1, Table S1: Search Strategy; Table S2: Reason for Exclusions; Table S3: Data Points Extracted; Table S4: Study Characteristics; Table S5.1 and S5.2: Patient Characteristics.
