# Supplementary material for: Clinical, Radiologic, and Functional Outcomes following Bone Grafting for Metacarpal Non-Unions: A Systematic Review
Source: J Clin Med. 2024 Feb 18;13(4):1148. doi: 10.3390/jcm13041148 (PMC10889921; doi:10.3390/jcm13041148)
Supplement: Supplementary file 1 [file jcm-13-01148-s001.zip › jcm-2831408-supplementary.pdf]

Table S1: Search Strategy

Table S1.1: EMBASE Search Strategy

Entry #	Query	EMBASE
**1**	Bone graft*.mp.	45132
**2**	exp bone matrix/	8280
**3**	exp bone morphogenetic protein/	41804
**4**	BMP.mp.	29640
**5**	exp bone transplantation/	51067
**6**	exp bone development/	108787
**7**	exp bone regeneration/	35316
**8**	Osteogenesis.mp.	32532
**9**	metacarpal ADJ3 non-union.mp.	5
**10**	metacarpal ADJ3 nonunion.mp.	8
**11**	metacarpal non union.mp.	2
**12**	exp metacarpal bone/	4790
**13**	metacarpus.mp	516
**14**	metacarpal ADJ3 fracture.mp.	1484
**15**	metacarpal.mp	9145
**16**	delayed union.mp.	2654
**17**	15 AND 16	21
**18**	1 OR 2 OR 3 OR 4 OR 5 OR 6 OR 7 OR 8	234706
**19**	9 OR 10 OR 11 OR 12 OR 13 OR 14 OR 17	6223
**20**	18 AND 19	737

Table S1.2: MEDLINE Search Strategy

Entry #	Query	MEDLINE
**1**	Bone graft*.mp.	31372
**2**	exp bone matrix/	4250
**3**	exp bone morphogenetic proteins/	22662
**4**	BMP.mp.	21600
**5**	exp bone transplantation/	33782
**6**	exp bone development/	71135
**7**	exp bone regeneration/	28020
**8**	exp osteogenesis/	41571
**9**	metacarpal ADJ3 non-union.mp.	4
**10**	metacarpal ADJ3 nonunion.mp.	7
**11**	metacarpal non union.mp.	2
**12**	exp metacarpal bones/	1947
**13**	exp metacarpus/	3855
**14**	metacarpal ADJ3 fracture.mp.	495
**15**	1 OR 2 OR 3 OR 4 OR 5 OR 6 OR 7 OR 8	161155
**16**	9 OR 10 OR 11 OR 12 OR 13 OR 14	5799
**17**	15 AND 16	578

Table S1.3: Cochrane Library Search Strategy

Entry #	Query	# of Results
**1**	bone graft*	6216
**2**	bone NEXT graft*	2578
**3**	MeSH [Bone Matrix]	156
**4**	MeSH [Bone Morphogenetic Proteins]	187
**5**	BMP	526

**6**	MeSH [Bone Transplantation]	1061
**7**	MeSH [Bone Development]	790
**8**	MeSH [Bone Regeneration]	895
**9**	MeSH [Osteogenesis]	367
**10**	metacarpal non-union	8
**11**	metacarpal nonunion	12
**12**	metacarpal non union	13
**13**	MeSH [Metacarpal Bones]	38
**14**	MeSH [Metacarpus]	35
**15**	metacarpal fracture	135
**16**	1 OR 2 OR 3 OR 4 OR 5 OR 6 OR 7 OR 8 OR 9	8072
**17**	10 OR 11 OR 12 OR 13 OR 14 OR 15	182
**18**	16 AND 17	6

Table S2: Reasons for Exclusion

**Author, year**	**Title**	**Reason for exclusion**
Aleksandrov et al., 2020	Reconstruction of fingers using skin-bone grafts with microvascular anastomoses	Wrong intervention
Brown, 1973	The management of phalangeal and metacarpal fractures	Wrong intervention
Bruner, 1957	Use of single iliac-bone graft to replace multiple metacarpal loss in dorsal injuries of the hand	Wrong intervention

Cherkashin, 1964	Replacement of metacarpal defects by the end section of the metatarsus	Other language
Chin and Vedder, 2008	MOS-PSSM CME article: Metacarpal fractures	Wrong intervention
Cooke et al., 1973	The fracture mechanics of bone – another look at composite modeling	Wrong population
Del Pinal and Innocenti., 2007	Evolving concepts in the management of the bone gap in the upper limb. Long and small defects	Review
D'Orio et al., 2023	A functional "metacarpal-hand" after a firework injury obtained without any flap or toe-transfer	Wrong intervention
Dy, 2020	What's New in Hand and Wrist Surgery	Wrong publication type
Ebrahimzadeh and Jupiter, 2007	Isolated Tuberculosis of a Metacarpal Bone in a 2-year-old child	Wrong publication type
Evans et al., 2008	The use free, mainly cancellous bone grafts for reconstruction of phalanges and metacarpal bones following tumour resection	Wrong intervention
Fallah et al., 2011	Miniplating of metacarpal fractures: An outcome study	Other language
Fontaine et al., 2010	Osseous vascular anatomy in the hand and wrist	Other language
Gajendran and Malone., 2015	Management of complications with hand fractures	Review
Gonzalez, 2007	Locked Intramedullary Nailing of Metacarpal Fractures Secondary to Gunshot Wounds	Wrong publication type
Gonzalez et al., 1993	Low-velocity gunshot wounds of the metacarpal: Treatment by early stable fixation and bone grafting	Wrong intervention
Hammert, 2011	Treatment of Nonunion and Malunion Following Hand Fractures	Review

Henneieng et al., 1962	Repair of loss of metacarpal substance with the aid of iliac grafts	Other language
Hsu et al., 2018	Chimeric medial femoral condyle osteocutaneous flap for reconstruction of multiple metacarpal defects	Review
Huffman and Rayan, 2011	Thumb metacarpophalangeal arthrodesis with local bone grafting	Wrong population
Innocenti et al., 2006	The use of allografts in hand surgery	Other language
Jaminet et al., 2010	Reconstruction of the second metacarpal bone with a free vascularized scapular bone flap combined with nonvascularized free osteocartilagenous grafts from both second toes: A Case Report	Wrong intervention
Jones Jr et al., 2012	Free vascularized medial femoral condyle autograft for challenging upper extremity nonunions	Review
Jupiter et al., 2007	Posttraumatic reconstruction in the hand	Wrong intervention
Kettelkamp, 1972	Experimental autologous joint transplantation	Wrong population
Kettlekamp and Ramsey, 1971	Experimental and clinical autogenous distal metacarpal reconstruction	Wrong population
Kolaityte et al., 2022	Intra-medullary Cannulated Headless Compression Screw for Bone Graft Fixation in Metacarpal Fractures	Wrong intervention
Kremer et al., 2006	Outcome Assessment after reconstruction of complex defects of the forearm and hand with osteocutaneous free flaps	Wrong intervention
Lee et al. 2000	Reconstruction of composite metacarpal defects using a fibula free flap	Wrong intervention

Moris et al., 2016	Functional and radiographic evaluation of the treatment of traumatic bone loss of the hand using the Masquelet technique	Wrong intervention
Murata, 2015	Vascularized bone grafts to the upper extremities	Full-text not found
Nanno et al., 2007	Dorsal fracture dislocations of the second and third carpometacarpal joints	Wrong publication type
Nathan and Chatterjee, 2005	Silastic replacement of metacarpal after resection of giant cell tumor. A case report	Wrong publication type
Omokawa, 2006	The anatomical basis for reverse first to fifth dorsal metacarpal arterial flaps	Wrong publication type
Picardo et al., 2021	Iliac crest bone grafting for metacarpal bone defects using bridging bone block	Wrong publication type
Poitevein, 2002	Trephine bone grafting technique	Wrong intervention
Ring, 2006	Malnunion and nonunion of the metacarpals and phalanges	Review
Rodriguez et al., 2022	Free medial femoral condyle flap for phalangeal and metacarpal bone reconstruction	Wrong intervention
Shipachev, 1958	Metacarpal bone graft in reconstruction of the fingers and hand	Other language
Singh et al., 2013	Percutaneous autologous bone marrow injections for delayed or non-union of bones	Wrong intervention
Soucacos et al., 2006	Vascularized bone grafts for the management of non-union	Review
Suematsu et al., 1987	Postoperative course of patients treated with iliac osteocutaneous free flaps. A two-to five-year follow-up study	Wrong intervention

Verega, 2017	Associated bone and soft tissue defects of the hand solved with vascularized bone plastys	Wrong intervention
Williams and Lochner, 2013	Pediatric hand and wrist injuries	Wrong intervention
Yajima et al., 1999	Free vascularized fibula grafts in surgery of the upper limb	Wrong intervention
Yong et al., 2007	Trapezoid rotational bone graft osteotomy for metacarpal and phalangeal fracture malunion	Wrong intervention

Table S3: Data Points Extracted

Category	Datapoint
**Study Characteristics**	– Article Title<br>– Author<br>– Year<br>– Country of publication<br>– Funding Source<br>– Methodology (Study type)
**Population Characteristics**	– Number of participants<br>– Sex<br>– Mean Age (range)<br>– Initial Injury<br>– Affected Metacarpal<br>– Time from injury until graft<br>– Number of prior bone operations<br>– Type of Nonunion (e.g., reactive, hypertrophic, etc.)<br>– Type of Bone Graft<br>– Graft vascularity
**Outcomes**	– Post-Op management<br>– Time to Follow-Up (in months) (range)

	– Time to Bone fusion/union (in months) (range)<br>– Rates of non-union/failure of bone graft<br>– Radiologic Outcomes<br>– VAS Score<br>– Post operative pain<br>– Range of motion (ROM)<br>– Grip strength (Kg)<br>– Pinch strength<br>– Complications<br>– Return to Activities of Daily Living (ADLs)<br>– Patient satisfaction<br>– Sensation<br>– Nonunion due to infection

Table S4: Study Characteristics

Study	Year	Study Type	Funding Source	Country	Purpose/Aim
Aguilera et al.	2022	Retrospective Case Series	None	Spain	Describe an original technique with a fixation method for the treatment of metacarpal non-union and report a series of three cases.
Akmaz et al.	2004	Prospective Cohort	None	Turkey	Evaluate the treatment approach and functional outcomes of surgical reconstruction performed on individuals who have sustained metacarpal shaft defects as a result of gunshot wounds.
Anderson et al.	2022	Case Report	None	USA	Act as an educational tool for the rising reconstructive surgeon explaining the ‘when, why and how’ for perioperative management.
Christen et al.	2022	Prospective Cohort	Yes	Switzerland	Investigate the osteogenic capacity, the advantages, and the outcome of free periosteal-only medial femoral condyle flaps in patients with metacarpal non-union with impaired bone vascularization.
Cogsil et al.	2022	Retrospective Case Series	None	USA	Describe a new surgical technique using an iliac crest "top hat" bone graft to treat refractory metacarpal for when initial surgical treatment of metacarpal non-union is unsuccessful.

Deng et Al.	2020	Case Report	None	China	Summarize the current application of vascularized small bone grafting for fracture non-union and bony defects.
Doi and Sakai	1994	Case Report	NR	Japan	Describes clinical applications of the vascularized periosteal bone graft to treat patients with fracture non-union.
Ebraheim et al.	1997	Case Report	NR	USA	Report the use of an AO mini fixator and autogenous iliac crest graft for segmental defects of the metacarpals.
Erçin et al.	2022	Retrospective Cohort Study	None	Turkey	Present adipofascial and periosteal tissue technical modifications and results for MFC free flap monitoring.
Ferguson and Bogoch	1999	Case Report	NR	Canada	Describe the case of an unusual seronegative monoarthropathy of the right wrist and who was treated with fusion by the method of Clayton.
Ireland and Taleisnkik	1986	Case Report	NR	USA	Present cases, review the literature on the subject, and discuss factors that contributed to the development of delayed and non-unions.
Jupiter et al.	1985	Retrospective Cohort Study	NR	USA	Review a consecutive series of 25 phalangeal and metacarpal non-union and delayed unions.

Milhoan et al.	2022	Case Report	None	USA	Present a technique for managing metacarpal bone loss utilizing a tri-cortical iliac crest graft and an intramedullary metacarpal nail for the treatment of metacarpal fractures and non-union.
Sakai et al.	1988	Case Report	NR	Japan	Describe a new vascularized thin corticoperiosteal graft harvested from the medial condylar and supracondylar areas of the femur.
Tsai et al.	1981	Case Report	NR	USA	Describe nine instances of combined second and third toe-to-hand transfers for severe transmetacarpal mutilating hand injuries.
Vegas et al.	2012	Prospective cohort	NR	Spain	Evaluate the efficacy of periosteal-only transfers from the medial femoral condyle in the treatment of recalcitrant non-unions.
Wei et al.	1999	Case Report	NR	Taiwan	Present experiences in bilateral metacarpal hand reconstruction with multiple-toe transplantations in a series of six patients
Zargarbashi et al.	2018	Case Report	None	Iran	Present a two-year-old boy with a non-union of metacarpal fractures, treated with open reduction/internal fixation and bone graft.

NR: Not reported

Table S5.1: Patient Characteristics

Study	N	Sex	Mean Age (Range)	Initial Injury	Initial Interventions	Affected Metacarpal	Time from injury
Aguilera et al. (2022)	4	M: 3<br>F: 1	44.5<br>(29-73)	M1: Oblique long comminuted and deviated fracture<br>M2: Short oblique fracture<br>M3: Rotated and shortened transverse fracture from the distal diaphysis<br>F1: long oblique fracture of the diaphysis	M1: ORIF, then cast<br>M2: Internal fixation<br>M3: ORIF<br>F: Conversative	M1: 3rd metacarpal<br>M2: 3rd metacarpal<br>M3: 4th metacarpal<br>F1: 5th metacarpal	M1: 3.5 m<br>M2: 6 m<br>M3: 4 m<br>F1: 5 m
Akmaz et al. (2004)	8	M: 8	22<br>(20-25)	Gunshot wounds	Debridement/skin closure	M1: 2nd and 3rd metacarpals<br>M2: 3rd metacarpal<br>M3: 3rd metacarpal<br>M4: 3rd metacarpal<br>M5: 3rd metacarpal<br>M6: 3rd metacarpal<br>M7: 3rd metacarpal<br>M8: 4th metacarpal	M1: 11 m<br>M2: 11 m<br>M3: 8 m<br>M4: 15 m<br>M5: 10 m<br>M6: 7 m<br>M7: 10 m<br>M8: 8 m
Anderson et al. (2022)	1	M: 1<br>F: 0	38	Mangled crush injury to the left hand	Debridement//pin insertion/K-wires, then debridement/skin graft, then debridement/K-wire removal	3rd metacarpal	7 m

Christen et al. (2022)	4	M: 2<br>F: 2	27.25<br>(16-32)	P1: Blast trauma, open<br>P2: Gunshot trauma, open<br>P3: Saw trauma, open<br>P4: Blunt trauma, closed	P1: ORIF/K-wire/cement<br>P2: ORIF/K-wire<br>P3: ORIF<br>P4: ORIF	P1: Diaphyseal, multifragmentary<br>P2: Diaphyseal, multifragmentary<br>P3: Metaphyseal, multifragmentary<br>P4: Diaphyseal, multifragmentary	P1: 8.8 m<br>P2: 2.13 m<br>P3: 9.96 m<br>P4: 16.63 m
Cogsil et al. (2022)	2	M: 1<br>F: 1	31.5<br>(31-32)	M: Traumatic work injury due to a bandsaw<br>F: Right thumb open fracture dislocation and degloving injury incurred using a wood splitter	M: Ray resection/finger transposition/plate, then reduction/plate fixation/bone grafting<br>F: Amputation, then osteotomy/unilateral external fixation, then ORIF/bone graft/plate	M: 3rd metacarpal<br>F: 1st metacarpal	M: 18 m<br>F: 14 m
Deng et Al. (2020)	1	M: 1<br>F: 0	31	Intra-articular fracture of the metacarpal head	Screw insertion/removal	4th metacarpal	14 m
Doi and Sakai (1994)	1	M: 1<br>F: 0	36	Open fracture	Plates/screws	Metacarpal shaft	NR
Ebraheim et al. (1997)	1	M: 1<br>F: 0	30	Displaced closed fracture of fourth metacarpal	ORIF/K-wires/tension-band wiring	5th metacarpal	2 m

Erçin et al. (2022)	11	M: 9<br>F: 2	35.5<br>(22-47)	Crush injury (M): 5<br>Gun injury (F): 2<br>Gun injury (M): 4	NR	NR	NR
Ferguson and Bogoch (1999)	1	M: 0<br>F: 1	36	Seronegative arthritis	Debridement, then wrist fusion, then Steinmann pin insertion/removal, then fiberglass splint, then debridement/T-plate fixation	3rd metacarpal	29 m
Ireland and Taleisnkik (1986)	1	M: 0<br>F: 1	10	Closed shaft fractures	Cast	2nd and 3rd metacarpals	7 m
Jupiter et al. (1985)	6	M: 6<br>F: 0	24.5<br>(20-31)	P1: Direct blow (closed midshaft fracture)<br>P2: Crush, degloving (open proximal 1/3 fracture)<br>P3: Rotary blade (open fracture)<br>P4: Direct blow (closed midshaft fracture)<br>P5: Crush, degloving (open base fracture)<br>P6: Direct blow (closed midshaft fracture)	P1: Splint<br>P2: ORIF/K-wires<br>P3: ORIF/K-wires x2<br>P4: ORIF/K-wires<br>P5: ORIF/K-wires x2<br>P6: ORIF	P1: 5th metacarpal<br>P2: 3rd metacarpal<br>P3: 4th metacarpal<br>P4: 2nd metacarpal<br>P5: 2nd and 3rd metacarpal<br>P6: 4th metacarpal	P1: 12 m<br>P2: 6 m<br>P3: 6 m<br>P4: 12 m<br>P5: 11 m<br>P6: 4 m

Milhoan et al. (2022)	1	M: 1<br>F: 0	19	Crush injury of previously treated gunshot wound	ORIF	4th metacarpal	13 m
Sakai et al. (1988)	1	M: 1<br>F: 0	36	Open fracture	Debridement/primary closure, then plates/screws, then curettage x3, then K-wires	2nd metacarpal	4 m
Tsai et al. (1981)	1	M: 1<br>F: 0	60	Degloving injury to the thumb and four fingers	Debridement/primary closure then phalanx/toe transplantation	1st and 5th MCP joint, 3rd and 4th through metacarpal base	28 m
Vegas et al. (2012)	1	M: 1<br>F: 0	37	NR	Internal fixation/bone graft	NR	NR
Wei et al. (1999)	1	M: 1<br>F: 0	21	Industrial injury	Stumped coverage using pedicled groin flaps, then toe transplantation with second-ray amputation	3rd metacarpal	7 m
Zargarbashi et al. (2018)	1	M: 1<br>F: 0	2	Crush injury	Fasciotomy, then splint x4 w	4th and 5th metacarpal	8 m

F: Female; M: Male; m: months; MCP: metacarpophalangeal; NR: Not Reported; N: number of participants; ORIF: open reduction and internal fixation; P: patient; w: weeks

Table S5.2: Patient Characteristics continued

Study	Type of Bone Graft	Graft Vascularity	# of Prior Bone Operations	Type of Non-union	Non-union due to infection
Aguilera et al. (2022)	M1: Autologous distal radius bone graft<br>M2: Distal radius bone graft<br>M3: NR<br>F: NR	NR	M1: 1<br>M2: 1<br>M3: 1<br>F1: 0	M1: Atrophic<br>M2: Hypertrophic<br>M3: NR<br>F1: NR	None
Akmaz et al. (2004)	Tricortical iliac bone graft	NR	1	NR	None
Anderson et al. (2022)	Autologous cancellous dorsal distal radius bone graft	NR	1	NR	None
Christen et al. (2022)	Cancellous bone graft + Autograft	Descending genicular artery: 3<br>Superomedial genicular artery: 1	NR	NR	None
Cogsil et al. (2022)	Iliac crest top hat bone graft	NR	M: 2<br>F: 2	NR	None
Deng et Al. (2020)	Osteochondral medal femoral condyle graft	Pedicle anastomosed end-to-end to the 2nd metacarpal artery	1	NR	None
Doi and Sakai (1994)	Vascularized corticoperiosteal graft	NR	4	NR	1/1
Ebraheim et al. (1997)	Cancellous iliac bone graft	NR	2	NR	1/1

Erçin et al. (2022)	Medial femoral condyle flap	Descending genicular artery	NR	NR	None
Ferguson and Bogoch (1999)	Failed small corticocancellous iliac crest bone graft, followed by 2 cm tricortical iliac crest bone graft	NR	2	Atrophic	None
Ireland and Taleisnkik (1986)	NR	NR	0	NR	None
Jupiter et al. (1985)	P1: Bone graft<br>P2: Iliac crest bone graft without internal fixation<br>P3: Bone graft<br>P4: Bone graft<br>P5: Autogenous iliac crest bone graft and K-wire<br>P6: Bone graft	NR	P1: 0<br>P2: 1<br>P3: 1<br>P4: 1<br>P5: 1<br>P6: 1	NR	1/6
Milhoan et al. (2022)	Tri-cortical iliac crest bone graft	NR	1	NR	None
Sakai et al. (1988)	Free vascularized corticoperiosteal graft from medial condylar and supracondylar areas of the femur	Dorsal branch of the radial artery and its vena comitans	4	Atrophic	1/1

Tsai et al. (1981)	NR	NR	2	NR	NR
Vegas et al. (2012)	Corticoperiosteal vascularized bone graft from medial femoral condyle	NR	1	Recalcitrant	NR
Wei et al. (1999)	Iliac bone graft	NR	3	NR	None
Zargarbashi et al. (2018)	Iliac crest intercalary bone graft	NR	0	Atrophic	None

F: Female; M: Male; NR: Not Reported; N: number of participants; P: patient
